# The Role of Oxidative Stress in the Pathogenesis and Treatment of Leishmaniasis: Impact on Drug Toxicity and Therapeutic Potential of Natural Products

**DOI:** 10.3390/toxics13030190

**Published:** 2025-03-07

**Authors:** Heliton Patrick Cordovil Brígido, Laís Gabrielly Abreu dos Santos, Renilson Castro de Barros, Juliana Correa-Barbosa, Paulo Victor Barbosa dos Santos, Rayana Franciele Lopes Paz, Amanda Ramos Pereira, Kelly Cristina Oliveira de Albuquerque, Marliane Batista Campos, Fernando Tobias Silveira, Sandro Percário, Maria Fâni Dolabela

**Affiliations:** 1 National Council for Scientific and Technological Development (CNPq), Federal University of Pará (UFPA), Belém 66075-110, PA, Brazil; 2 Biotechnology and Biodiversity Postgraduate Program (BIONORTE), Federal University of Pará, Belém 66075-110, PA, Brazil; ams.ramos@outlook.com (A.R.P.); kellyoalbuquerque@gmail.com (K.C.O.d.A.); percario@ufpa.br (S.P.); 3 Faculty of Pharmacy, Federal University of Pará, Belém 66075-110, PA, Brazil; laisgabriellyas@gmail.com (L.G.A.d.S.); paulo.victor@ics.ufpa.br (P.V.B.d.S.); rayanaaapaz@gmail.com (R.F.L.P.); 4 Pharmaceutical Sciences Postgraduate Program, Federal University of Pará, Belém 66075-110, PA, Brazil; renilsonbarros098@gmail.com; 5 Postgraduate Pharmaceutical Innovation Program, Institute of Health Sciences, Federal University of Pará, Belém 66075-110, PA, Brazil; correabjuliana@gmail.com; 6 Parasitology Department, Evandro Chagas Institute, Ananindeua 67030-000, PA, Brazil; marlianecampos@iec.gov.br (M.B.C.); fernandotobias@iec.pa.gov.br (F.T.S.)

**Keywords:** leishmaniasis, oxidative stress, natural products

## Abstract

The treatment of leishmaniasis has limitations due to drug toxicity and the increasing resistance of the parasite. In this study, we analyze the role of oxidative stress in the pathogenesis and treatment of leishmaniasis, as well as in new therapeutic alternatives of natural origin. The evasion mechanisms against the host immune response involve surface molecules present in the parasite, which modulate oxidative stress to ensure its survival. Drug treatment requires strict monitoring to minimize adverse reactions and ensure patient safety, as mechanisms such as lipid peroxidation, mitochondrial dysfunction, and depletion of antioxidant defenses are associated with drug toxicity. Plant-derived products with antileishmanial activity impact the parasite’s redox balance, inducing apoptosis and reducing its parasitic load. Most studies are still in preliminary stages, making in vivo assays and clinical studies essential, along with the development of accessible formulations. Oxidative stress is involved in the pathogenesis of leishmaniasis, as *Leishmania* manipulates the host’s redox balance to survive. It also contributes to drug toxicity, as antimonials and amphotericin B increase reactive oxygen species, causing cellular damage. Several plant-derived compounds have demonstrated antileishmanial activity by modulating oxidative stress and promoting parasite apoptosis. Examples include alkaloids from *Aspidosperma nitidum*, lignans from *Virola surinamensis*, flavonoids from *Geissospermum vellosii*, and triterpenoids such as β-sitosterol. Although these compounds show promising selectivity, most studies remain in preliminary stages, requiring in vivo assays and clinical studies to confirm efficacy and safety, as well as the development of affordable formulations.

## 1. Introduction

Leishmaniasis is one of the ten leading neglected tropical diseases, representing a serious global public health problem. The disease is present in various regions of the world, including the Americas, East Africa, North Africa, and West and Southeast Asia. It is estimated that over 12 million people are infected, with the disease being endemic in 99 countries. Specifically, cutaneous leishmaniasis (CL) is endemic in 89 countries, while visceral leishmaniasis (VL) occurs in 80 countries. Additionally, 71 countries have both clinical forms of the disease as endemic, highlighting its wide geographical distribution and impact on public health [1].

The etiological agent of leishmaniasis is a parasite of the genus *Leishmania*, belonging to the family *Trypanosomatidae*, which includes approximately 22 species pathogenic to humans. These species are classified into the subgenera *Leishmania* and *Viannia*. The parasite has a digenetic life cycle, alternating between two morphological forms: the promastigote form, found in the phlebotomine vector, and the amastigote form, which develops inside the cells of the vertebrate host [2].

Clinically, leishmaniasis manifests in different forms and is classified into three main categories: cutaneous, mucosal/mucocutaneous, and visceral. Visceral leishmaniasis, the most severe form of the disease, is characterized by weight loss, hepatosplenomegaly, and anemia, which can lead to death in over 90% of untreated cases. Mucosal/mucocutaneous leishmaniasis affects the mucous membranes of the nose and mouth, potentially causing severe tissue destruction and disability. Cutaneous leishmaniasis, the most common form, causes ulcerative skin lesions, leaving permanent scars and impacting patients’ quality of life [3].

Given the complexity of the disease and the associated therapeutic challenges, this study analyzes the infection mechanisms of the parasite in vertebrate hosts, exploring the role of oxidative stress in the pathogenesis and treatment of leishmaniasis. Additionally, new therapeutic approaches of natural origin are discussed, with emphasis on plant-derived compounds, such as *Aspidosperma nitidum*, *Virola surinamensis*, *Geissospermum vellosii*, and *Corchorus capsularis*, which have demonstrated antileishmanial activity. These natural products modulate oxidative stress, promoting parasite apoptosis and exhibiting low cytotoxicity to host cells. The study evaluates the role of oxidative stress in both the efficacy and toxicity of these alternatives, aiming to contribute to the development of safer and more effective strategies to combat the disease.

## 2. Human Infection by the *Leishmania* Parasite, Immune Response, and Oxidative Stress

Parasites of the genus *Leishmania* have developed various adaptation mechanisms to ensure their survival in hostile environments throughout their life cycle [4]. In the vertebrate host, the first barrier faced by the parasite is the complement system, which plays a role in defending against foreign agents. However, *Leishmania* uses mechanisms to avoid its destruction by the complement system. Lipophosphoglycan (LPG), a glycoconjugate present on the surface of the parasite, prevents the insertion of the membrane attack complex (C5b-9), protecting the cell against lysis. In addition, the glycoprotein gp63 plays an important role in immune evasion by cleaving the C3b molecule into C3bi. This would prevent the formation of C5 convertase, activating the complement cascade and allowing the parasite to avoid elimination by the immune system [5].

Once they escape extracellular destruction, the parasites are phagocytosed by macrophages, specialized cells of the immune system. This process occurs mainly through the interaction of C3b and C3bi molecules with specific receptors, such as CR1 and CR3, present on the surface of macrophages. The internalization of the parasite via CR3 represents an efficient immune evasion strategy, as it prevents the activation of the microbicidal respiratory burst and reduces the production of IL-12, a cytokine essential for the immune response against infections [5].

In addition to interacting with complement receptors, metacyclic promastigotes can be opsonized by immunoglobulins and internalized via Fc receptors. Another phagocytosis pathway involves an interaction between LPG molecules and the mannose receptor on macrophages [4]. GPL also binds to C-reactive protein (CRP), one of the first molecules involved in the inflammatory response, promoting the parasite’s entry through CRP receptors [6]. Furthermore, both gp63 and GLP can interact with fibronectin and complement receptor 4 (CR4), expanding the possibilities of Leishmania internalization [7].

Once inside macrophages, the parasite is endocytosed into a phagosome, which subsequently undergoes a series of fusions to form the phagolysosome. In this environment, the amastigote form becomes susceptible to acidic and hydrolytic degradation promoted by the phagolysosome. However, *Leishmania* can modulate this process by influencing intracellular calcium levels and inhibiting protein kinase C (PKC) activity, thereby preventing the activation of the destruction mechanism [8,9].

In addition to macrophages, other phagocytic cells in the skin can be targeted for infection, including Langerhans cells (LCs), which express receptors for the C3 component of the complement system [10]. LCs play a crucial role in American cutaneous leishmaniasis (ACL), as they are responsible for presenting parasitic antigens to T lymphocytes in regional lymph nodes [11,12,13]. The surface of these cells contains essential molecules for the immune response, including MHC II, Fc and C3b receptors, ICAM-1, ICAM-3, and CD1, as well as IL-2 receptors and membrane ATPase activity [12]. Thus, LCs are essential in initiating the immune response against *Leishmania*, promoting the differential activation of CD4 T cells, Th1, and Th2. The balance between these different response profiles directly influences the host’s ability to eliminate or tolerate the parasite, with the Th1 response stimulating leishmanicidal mechanisms through the production of IL-2, IFN-γ, and TNF-α, while the Th2 response promotes parasite persistence by inducing immunosuppressive cytokines such as IL-4, IL-5, IL-6, IL-10, and IL-13 [14].

*Leishmania* infection can modulate the innate immune response, leading to a state of immunosuppression. Langerhans cells, for example, when presenting parasitic antigens via MHC II to CD4 T cells, can inhibit inflammatory events and favor parasite evasion [15]. Clinical studies have found that in patients infected with *L.* (*L.*) *amazonensis*, there is a progressive increase in the density of Langerhans cells compared to the reduction of CD4 and CD8 cells, indicating a modulation of the immune response in favor of the parasite [16].

The immune response mediated by CD4 Th1 T cells plays a crucial role in activating macrophages for parasite elimination. This process occurs through the production of nitric oxide (NO) by the enzyme nitric oxide synthase type 2 (NOS2), one of the main leishmanicidal mechanisms in murine macrophages and canine infections [17,18]. Additionally, in monocytes of infected dogs and in polymorphonuclear cells from peripheral blood, the production of the superoxide anion also significantly contributes to parasite killing [19].

**Figure 1:**
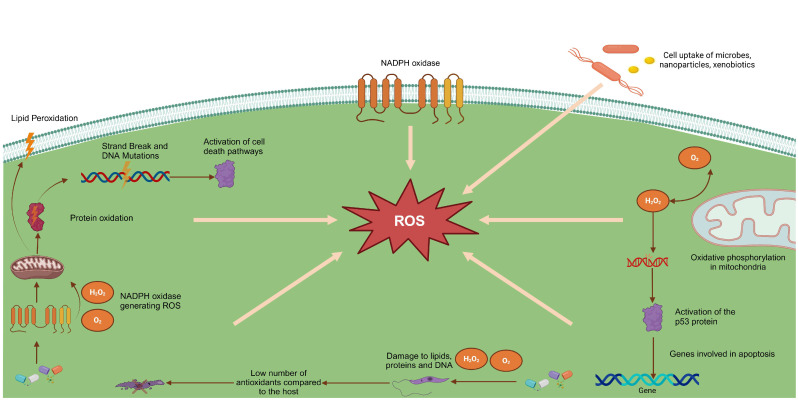
Representation of the impact of reactive oxygen species (ROS) on the host cell and the *Leishmania* parasite.

Figure 1 illustrates the effects of reactive oxygen species (ROS) generated during an immune response, highlighting their impact on various cellular structures. The activation of NADPH oxidase in cell membranes leads to the production of superoxide anions (O_2_^−^) and hydrogen peroxide (H_2_O_2_), which are crucial for parasite killing. These free radicals induce significant damage, such as lipid peroxidation, protein oxidation, and DNA strand breaks, in addition to activating apoptotic pathways and the p53 protein, triggering programmed cell death.

However, *Leishmania* has developed various strategies to resist the oxidative stress generated by macrophages. The parasite can interfere with iNOS induction and reduce the response to IFN-γ, as well as inhibit the oxidative burst by modulating PKC activity [20,21]. To protect itself from ROS and reactive nitrogen species (RNS), *Leishmania* utilizes molecules such as glutathione, trypanothione, and ovothiol A, which act as non-enzymatic scavengers of free radicals [22,23,24].

Tryparedoxin, for example, transfers reducing equivalents from trypanothione to tryparedoxin peroxidase (TXNPx) or to a glutathione peroxidase homolog, aiding in the neutralization of oxidative stress. Interestingly, *Leishmania* lacks catalase or a classical glutathione peroxidase, making other antioxidant mechanisms essential for its survival [25,26]. Additionally, iron superoxide dismutase (FeSOD) plays a crucial role in eliminating superoxide toxicity [27,28]. GLP also contributes to oxidative stress resistance by scavenging oxygen radicals [29], as well as by preventing the assembly of NADPH oxidase in the phagolysosome [30]. Additionally, a cellular chaperone HSP70 has been identified as a protective factor against oxidative stress [31].

In this way, *Leishmania* demonstrates a remarkable ability to adapt, escaping the immune response and modulating the intracellular environment to favor its survival. The balance between the host’s immune mechanisms and the parasite’s evasion strategies defines the progression of the disease and the effectiveness of potential therapeutic approaches. In this context, oxidative stress plays a crucial role, both in the elimination of the parasite by the immune system and in its resistance and persistence in the host. While macrophages trigger ROS and RNS to destroy the parasite, *Leishmania* develops efficient antioxidant mechanisms to neutralize these compounds and ensure its survival. Furthermore, the redox imbalance induced by antileishmanial drugs can be explored as a therapeutic strategy, enhancing the parasite’s vulnerability and promoting its cell death. Therefore, understanding the impacts of oxidative stress on the parasite–host interaction is essential for the development of new, more effective, and selective therapies.

## 3. Treatment of Leishmaniasis and Possible Involvement of Oxidative Stress

The treatment of leishmaniasis is complex and depends on multiple factors, including the clinical form of the disease, the presence of comorbidities, the species of the parasite involved, and the geographical location of the patient. Although it is a treatable and curable disease, the effectiveness of therapy is directly related to the competence of the immune system, as the available drugs do not completely eradicate the parasite. Therefore, immunocompromised patients have an increased risk of relapses and complications [32].

Among the first-line therapeutic options, pentavalent antimonials stand out, represented by meglumine antimoniate (Glucantime^®^) and sodium stibogluconate. These compounds have similar mechanisms of action, with their efficacy and toxicity being directly influenced by the antimony content in their formulation. The most common side effects include gastrointestinal symptoms, such as anorexia, vomiting, nausea, and abdominal pain, as well as systemic manifestations like malaise, myalgia, arthralgia, headache, metallic taste, and lethargy [33].

In addition to these symptoms, antimonials can induce electrocardiographic changes, with the most common being T wave inversion, QT interval prolongation, and the occurrence of arrhythmias. Elevated levels of pancreatic and hepatic enzymes are also common, as well as blood dyscrasias such as leukopenia, anemia, and thrombocytopenia [1].

Meglumine antimoniate (MA) caused significant protein carbonylation in the heart, spleen, and brain tissue. Increased lipoperoxidation was found in the liver and brain tissue. An imbalance between the activities of superoxide dismutase and catalase can be observed in the heart, liver, spleen, and brain tissue, suggesting that MA induces oxidative stress in several vital organs. This indicates that the production of highly reactive oxygen and nitrogen species induced by MA may be involved in some of its toxic effects [34].

Given the challenges posed by the toxicity of conventional treatments, new approaches have been explored. Recent studies investigated the potential of green-synthesized zinc nanoparticles (ZnNPs) in combating *Leishmania major*, either alone or in combination with meglumine antimoniate (MA). The combination of ZnNPs + MA resulted in a significant synergistic effect, reducing the 50% inhibitory concentration (IC_50_) to 12.6 µg/mL, compared to 43.2 µg/mL for ZnNPs alone and 26.3 µg/mL for MA [35].

Treatment with ZnNPs modulated the immune response in a favorable manner, promoting the increased expression of iNOS, TNF-α, and IFN-γ while reducing levels of IL-10, a cytokine associated with the parasite’s immune evasion. Additionally, significant activation of caspase-3 was observed, indicating a potential role in inducing apoptosis in parasitized cells without causing significant toxicity to normal cells [35].

Another drug widely used in the treatment of leishmaniasis is amphotericin B, which can be administered in its conventional or liposomal form. Although effective, its administration is associated with significant adverse reactions, such as high fever, stiffness, chills, and thrombophlebitis. Moreover, more severe effects include nephrotoxicity, hypokalemia, and myocarditis [36].

The mechanism of action of amphotericin B involves binding to ergosterol present in the cell membrane of fungi and protozoa, leading to destabilization and cell lysis [37]. However, this interaction is not completely selective and can also occur with cholesterol in human cells, which contributes to its toxicity [36]. The nephrotoxicity induced by the drug has been associated with the excessive generation of reactive oxygen species (ROS), which cause mitochondrial damage and promote the activation of exaggerated inflammatory responses, intensifying tissue injury [38].

Paromomycin, another antileishmanial agent, when administered, can cause mild pain at the injection site, as well as ototoxicity, renal toxicity, and hepatotoxicity [39]. Auditory toxicity, in particular, has been associated with the accumulation of reactive oxygen species (ROS), which promote apoptosis and irreversible damage to the cells of the inner ear [40]. Additionally, mechanisms such as lipid peroxidation, mitochondrial dysfunction, and the depletion of cellular antioxidant defenses contribute to the toxicity of this drug [41].

Another drug used, pentamidine isetionate, is related to oxidative stress, the inhibition of nucleic acid synthesis, and mitochondrial dysfunction [42]. The accumulation of ROS in parasite cells can lead to mitochondrial dysfunction, ATP depletion, and programmed cell death [43]. In the host, pentamidine can cause nephrotoxicity, and it may also potentially contribute to the development of diabetes by inducing the destruction of pancreatic beta cells [44]. Additionally, its cardiac toxicity, characterized by arrhythmias and QT interval prolongation, may be related to oxidative damage in myocardial cells [45].

Miltefosine, one of the few orally available drugs, is associated with gastrointestinal adverse events, including anorexia, nausea, vomiting (38%), and diarrhea (20%). Additionally, it has teratogenic potential, being contraindicated for pregnant women and women with reproductive potential [46,47]. Its mechanism of action appears to involve the induction of oxidative stress both in the parasite and in the host cells, which may contribute to its therapeutic effects but also to its toxicity [48].

Mitochondria are one of the main targets of miltefosine toxicity. The excessive accumulation of ROS in these organelles can lead to mitochondrial dysfunction and cell apoptosis, particularly in susceptible tissues such as the kidneys and liver. Furthermore, there is evidence that the oxidative stress induced by the drug can cause DNA damage, resulting in strand breaks, mutations, and cell death, which may be related to its teratogenic and embryotoxic effects [49].

The parenteral administration of antileishmanial drugs requires strict monitoring to minimize adverse events and ensure the safety of the treatment. However, this need significantly increases therapeutic costs and complicates patient adherence, especially for those living in remote areas. Moreover, the growing resistance of the parasite to conventional drugs highlights the urgency of finding new effective therapeutic alternatives against resistant strains [50].

Finally, it is important to highlight the dual role of oxidative stress in leishmaniasis. While it plays an essential role in the destruction of the parasite by host cells, the same phenomenon, when induced by available drugs, can be directly associated with their toxic effects. Given this scenario, a central question arises: do natural products with leishmanicidal activity act through the induction of oxidative stress, or alternatively, do they follow a distinct pathway, thereby reducing the risk of toxicity?

## 4. New Therapeutic Alternatives for the Treatment of Leishmaniasis and Their Toxic Potential

The search for new therapeutic approaches against leishmaniasis has led to the investigation of plant extracts, fractions, and isolated compounds with antileishmanial potential. While some studies have been limited to in vitro assays, others have progressed to in vivo models, allowing for a more comprehensive evaluation of the efficacy and safety of these substances. However, the mechanisms of action of these compounds, particularly regarding the induction of oxidative stress, are not yet fully understood, nor are the data on their toxicity.

One study evaluated the antileishmanial activity of extracts obtained from the leaves of *Virola surinamensis* against *L. chagasi* and *L. amazonensis*. The ethyl acetate, methanol extracts, and fractions C1–C6 did not show activity against promastigotes and did not have a significant effect on *L. amazonensis* amastigotes. The hexane extract (HEVS) was the only one to exhibit activity against *L. chagasi* promastigotes (IC_50_ = 86.40 µg/mL) and *L. amazonensis* (IC_50_ = 79.7 ± 1.3 µg/mL), and it was considered active. However, the fraction and surinamesine did not show a significant increase in antipromastigote activity compared to the crude extract [51].

Moreover, cytotoxicity tests demonstrated that all the samples tested exhibited low cellular toxicity (CC_50_ > 500 µg/mL), resulting in selectivity indices (SI) greater than 5.8 for *L. chagasi* and 6.2 for *L. amazonensis*, indicating a favorable safety profile. However, the extracts did not show any effect on intracellular amastigote forms of *L. amazonensis*, suggesting the need for structural modifications or formulations that could improve their in vivo efficacy (Table 1) [51].

Another investigation analyzed the action of (-)-5-desmethoxygrandisine B, a compound isolated from *V. surinamensis*, against promastigotes and intracellular amastigotes of *L. amazonensis*. Treatment with this lignan induced ultrastructural alterations in promastigotes, such as mitochondrial swelling, kDNA disorganization, vacuole formation, vesicular structures, and an increase in flagellar pockets. Additionally, it reduced mitochondrial membrane potential and interacted with critical residues of the trypanothione reductase enzyme (TryR), suggesting that its leishmanicidal action may be related to the induction of oxidative stress (Table 1) [52].

The antiparasitic activity of β-sitosterol, a compound isolated from the chloroform extract of *Corchorus capsularis* L. and present in various plant species, showed efficacy against *L. donovani* promastigotes (IC_50_ = 17.7 ± 0.43 µg/mL) and induced oxidative stress in the parasite, leading to intracellular ROS production. As a consequence, cell apoptosis was observed, characterized by mitochondrial membrane depolarization, phosphatidylserine externalization, and DNA fragmentation. Molecular docking studies indicated that β-sitosterol inhibited trypanothione reductase (LdTryR), an essential enzyme for the parasite’s redox balance (Table 1) [53].

Similarly, the methanolic extract obtained from the bark of *Sterculia villosa* Roxb. (SVE) exhibited concentration-dependent antipromastigote activity against *L. donovani* (IC_50_ = 17.5 µg/mL). Treatment resulted in increased levels of ROS, superoxide, and lipid peroxidation, as well as DNA fragmentation, suggesting a mechanism of action based on oxidative stress induction. It is important to note that the extract did not show significant cytotoxicity, indicating a favorable safety profile (Table 1) [54].

Another study investigated the effect of flavopeirerine, an alkaloid isolated from *Geissospermum vellosii*, against *L. amazonensis*. Fractionation of the extract contributed to increased antipromastigote activity, with flavopeirerine showing high leishmanicidal potency, with IC_50_ values of 0.23 µg/mL (24 h) and 0.15 µg/mL (72 h). Selectivity was also specific, presenting a selectivity index (SI) of 976.2 (24 h) and 4993.2 (72 h), demonstrating greater safety compared to amphotericin B, the therapeutic reference for leishmaniasis (Table 1) [55].

Molecular dynamics studies revealed beneficial and selective interactions with the parasite’s oligopeptidase B (OpB), an enzyme associated with *Leishmania* virulence and adaptation to oxidative stress. Flavopeirerine interacted with the Tyr-499 residue of OpB, indicating an inhibitory effect on this enzyme. Since OpB is involved in regulating proteins essential for the parasite’s antioxidant defense, its inhibition may impair *Leishmania*’s ability to neutralize ROS generated by the host immune system. This mechanism may enhance the leishmanicidal effect of flavopeirerine, making the parasite more susceptible to oxidative stress and reducing its ability to replicate and survive (Table 1) [55].

Among the investigated species, *Aspidosperma nitidum* has emerged as a promising candidate for leishmaniasis treatment. Four recent studies have evaluated its efficacy and toxicity in vitro and in vivo [56,57,58,59]. The first study evaluated the in vitro leishmanicidal activity of extracts and fractions of *A. nitidum* against *L. amazonensis*. The results showed that the ethanolic extract (EE) exhibited significant activity against intracellular amastigotes with an IC_50_ of 23.87 µg/mL, while the alkaloid fraction (AF) displayed a lower IC_50_ of 18.5 µg/mL, indicating greater antileishmanial potency. The dichloromethane fraction (FrDCL) demonstrated moderate activity against promastigotes (IC_50_ = 105.7 µg/mL), revealing higher selectivity for the intracellular form of the parasite. The relationship between leishmanicidal activity and cytotoxicity showed that EE had a SI of 21, while AF had an SI of 11, reinforcing the therapeutic potential of this species (Table 1) [57].

The difference in selectivity indices (SIs) between extracts may be related to the chemical composition of each fraction [56]. While the ethanolic extract (EE) presents a greater diversity of bioactive compounds, the alkaloid fraction (AF) may contain substances with more potent action but with greater cytotoxicity. This variation reinforces the importance of specific chemical characterization for the selection of compounds with a better therapeutic profile.

The second study identified that alkaloids isolated from this plant induced morphological alterations in *L. amazonensis*, including flagellum shortening, cell rounding, and the formation of cytoplasmic vacuoles, suggesting a mechanism of action related to mitochondrial dysfunction and oxidative stress (Table 1) [58].

Another study evaluated the acute and subacute toxicity of *A. nitidum* extracts in BALB/c mice. No clinical, metabolic, or histopathological alterations were observed after the administration of 2000 mg/kg in the acute phase and 1000 mg/kg over 28 days in the subacute phase, indicating a favorable safety profile compared to conventional drugs that often induce oxidative stress and hepatorenal toxicity (Table 1) [56].

The in vivo efficacy of *A. nitidum* extracts was also evaluated in mice infected with *L. amazonensis*. The treatment significantly reduced the splenic parasite load and modulated the immune response by promoting an increase in IFN-γ production and a decrease in IL-10 levels, a cytokine associated with immunosuppression. Additionally, histopathological analysis revealed an accelerated healing process, with reduced parasitic infiltration and increased collagen fiber deposition, indicating a regenerative effect associated with the treatment (Table 1) [59].

The investigation of the mechanism of action of *A. nitidum* alkaloids indicated that corynantheol, yohimbine, and dihydrocorynantheol act as potential inhibitors of trypanothione reductase (TR), a critical enzyme for *Leishmania*’s antioxidant defense. Molecular docking experiments demonstrated favorable interactions, and molecular dynamics simulations confirmed the stability of these interactions, suggesting strong enzymatic inhibition. Since *Leishmania* lacks glutathione reductase, TR inhibition compromises ROS neutralization, leading to cellular dysfunction and parasite death. This mechanism represents a significant advantage over conventional drugs, such as antimonials and amphotericin B, which induce oxidative stress nonspecifically and may cause systemic toxicity (Table 1) [59].

Studies indicate that anthracene endoperoxides (AcEPs) AcEP1117, AcEP1118, AcEP1129, and AcEP1130, derived from *Artemisia annua*, exhibit significant activity against *Leishmania*, with IC_50_ values in the low micromolar range. AcEP1118 and AcEP1129 were the most potent compounds, showing IC_50_ values of 1.00 ± 0.73 µM and 0.61 ± 0.21 µM against *L. tarentolae* promastigotes, respectively. For *L. donovani* promastigotes, AcEP1117 exhibited an IC_50_ of 2.65 ± 0.34 µM, while AcEP1129 and AcEP1130 showed IC_50_ values of 4.21 ± 0.36 µM and 39.48 ± 3.48 µM, respectively [60].

The investigation of the interaction kinetics between these compounds and iron revealed the formation of oxygen- and carbon-centered radicals, which trigger the secondary production of superoxide radicals, compromising the parasite’s mitochondrial functions and leading to its cell death. Since iron is essential for *Leishmania* replication, participating in metabolic processes and DNA biosynthesis, the parasite’s dependence on this metal becomes a strategic target for new therapies. However, it was observed that AcEPs also exhibit toxicity toward J774 macrophages, with IC_50_ values ranging from 0.90 ± 0.28 µM (AcEP1129) to 43.80 ± 36.12 µM (AcEP1117), which may limit their therapeutic use without structural modifications to enhance selectivity [60].

Aiming to optimize efficacy and reduce toxicity, one of the artemisinin analogs, artemether (ART), was incorporated into a lipid-based nanostructure (NLC-ART) and tested against *Leishmania infantum*. Both ART and NLC-ART were active against promastigote forms (IC_50_ ART = 37.12 µg/mL; NLC-ART = 32.1 µg/mL) and amastigote forms (IC_50_ ART = 16.43 µg/mL; NLC-ART = 15.42 µg/mL), with a slight reduction in IC_50_ values for the nanostructured formulation. This strategy may represent a significant advancement in drug targeting to the parasite, reducing systemic toxicity and increasing efficacy (Table 1) [61].

In addition to endoperoxides, phenylpropanoid glycosides, such as salidroside (SAL), have been studied for their immunomodulatory and antileishmanial properties. Isolated from species of the *Rhodiola* genus, SAL is known for its protective activity in various physiological systems, including the liver [62], heart [63], and nervous system [64]. In addition, it has shown therapeutic potential for inflammatory skin diseases [65].

The leishmanicidal activity of SAL was evidenced by its ability to interrupt the cell cycle of *L. donovani*, halting the promastigotes at the sub-G0/G1 stage [66]. In a murine model of visceral leishmaniasis, treatment with SAL significantly reduced the parasitic load. In addition to modulating the immune response and promoting a greater polarization toward the Th1 profile, the compound stimulated the production of CD4+ and CD8+ T cells, favoring a more effective response against the parasite. Molecular analysis showed increased expression of genes related to oxidative stress, such as NF-ĸB, iNOS, NO, and ROS. It is important to note that SAL showed minimal toxicity to human THP-1 cells and did not reveal toxic effects on the liver and kidneys (Table 1) [66].

Another group of promising compounds are the iridoid glycosides isolated from *Nyctanthes arbortristis*, which have the ability to induce oxidative stress in the parasite. Among the compounds evaluated, arbortristoside A, arbortristoside B, and arbortristoside C showed significant leishmanicidal activity against *L. donovani*, promoting an increase in ROS production and an imbalance in the parasite’s redox homeostasis. These compounds act by inhibiting TR, an enzyme crucial for the antioxidant defense of *Leishmania*. The reduction in reduced thiol levels limits the parasite’s ability to neutralize oxidative stress, causing progressive cellular damage and apoptosis. In vitro tests showed that the iridoid glycosides demonstrated high potency against intracellular amastigotes, even at low concentrations. Furthermore, toxicity evaluation in human embryonic kidney cells (HEK 293) and mouse macrophages (J774A.1) indicated that these compounds have low cytotoxicity, revealing a favorable safety profile (Table 1) [67].

HO-3867, a curcumin analog belonging to the class of diarylidenylpiperidones (DAPs), is a promising inhibitor of *Leishmania* metabolism. This compound is a potent inhibitor of the STAT3 signaling pathway, acting in the modulation of inflammation and cellular apoptosis. Furthermore, it has the ability to induce ROS production, activate caspase-3, and promote PARP1-mediated apoptosis, a mechanism previously observed in cancer cells (Table 1) [68,69,70,71,72].

Despite their therapeutic potential, curcuminoids face challenges related to their low bioavailability, which limits their clinical efficacy [73,74]. To overcome this limitation, a liposomal formulation of HO-3867 (PC-SA/HO-3867) was developed, which was shown to increase the stability and absorption of the drug [75].

In vitro assays revealed that PC-SA/HO-3867 induced apoptosis in *L. donovani*, evidenced by changes in cell morphology, the externalization of phosphatidylserine, mitochondrial depolarization, and the accumulation of intracellular lipids. Additionally, the compound caused cell cycle arrest in promastigotes and significantly reduced the load of intracellular amastigotes. Regarding safety, it exhibited low cytotoxicity to murine macrophages [75].

Additionally, treatment with PC-SA/HO-3867 induced the activation of metacaspase and PARP1 in *L. donovani*, as well as negatively regulating the expression of the Sir2 gene, involved in the parasite’s longevity. This formulation also reduced the intracellular load of *L. donovani* amastigotes in in vitro assays. The results suggest that this compound exerts its action through the induction of oxidative stress and increased NO production, enhancing the parasite’s susceptibility to immune-mediated destruction (Table 1) [75].

Among the compounds developed, flavopeirine, β-sitosterol, and alkaloids from *A. nitidum* stood out due to their high selectivity index. These compounds represent promising candidates for the development of new antileishmanial therapies, as they showed significant leishmanicidal activity with low toxicity to host cells.

Thus, different natural and synthetic compounds have shown therapeutic potential against leishmaniasis in various studies, many of them exploring the selective induction of oxidative stress as a mechanism of action. However, the transition of these candidates to clinical use requires additional studies on pharmacokinetics, chronic toxicity, and clinical trials to confirm their safety and efficacy in humans.

## 5. Conclusions

Oxidative stress plays a crucial role in the immune response and *Leishmania* infection. During immune activation, macrophages present various ROS and NO to eliminate the parasite. However, *Leishmania* has developed mechanisms to neutralize this oxidative stress, ensuring its intracellular survival. The imbalance between ROS production and neutralization can influence the progression of the infection and the effectiveness of the host’s immune response.

Furthermore, oxidative stress is strongly associated with the toxicity of drugs used in the treatment of leishmaniasis. Drugs such as antimonials, amphotericin B, and miltefosine induce excessive ROS generation, resulting in mitochondrial damage, cellular apoptosis, and tissue inflammation. These adverse effects compromise the safety of the treatment and limit its applicability, requiring rigorous monitoring to minimize systemic toxicity.

This research highlights that most studies on new therapeutic alternatives for leishmaniasis are still in the preliminary stage and are predominantly conducted in vitro. Although these studies provide important insights into the mechanisms of action and toxicity of the compounds investigated, there is still a significant gap in understanding the benefits and risks of oxidative stress induced by plant-derived substances and their derivatives. Additionally, the development of specific formulations, while promising, presents challenges related to high costs, which may limit patient access to these treatments.

In light of these findings, it is essential to establish strict guidelines for the research and development of new leishmanicidal drugs. In oral toxicity studies, it is necessary to evaluate whether the substances under testing induce oxidative changes and possess immunomodulatory properties in healthy animals. Then, in experimental models of *Leishmania* infection, it is important to investigate whether oxidative modifications occur and how these interactions influence the immune response, comparing these effects with positive and healthy control groups.

In addition to antiparasitic efficacy, the development of accessible formulations should be prioritized. Strategies to enable low-cost oral formulations are crucial to expanding the availability of these treatments, especially in endemic regions where leishmaniasis represents a serious public health problem.

Finally, the continuation of studies should encompass an integrated approach, considering not only the leishmanicidal activity of the compounds but also their safety, economic forecast, and potential for clinical use. Only through this multidimensional approach will it be possible to develop innovative therapies that are effective, safe, and accessible, contributing to the control and treatment of leishmaniasis in a sustainable and equitable manner.

## Figures and Tables

**Table 1:** Therapeutic and toxicological profile of alternatives in the treatment of leishmaniasis.

Samples	Antileishmania Activity		Toxicity Profile	SI	Mechanism of Action
	Promastigote	Amastigote			
*Virola surinamensis* (hexanic extract)	IC_50_ = 86.40 µg/mL (*L. chagasi*), 79.7 ± 1.3 µg/mL (*L. amazonensis*)	Inactive	(CC_50_ > 500 µg/mL) Low toxicity	>5.78 >6.27	Modulation of oxidative stress and apoptosis in the parasite
(-)-5-Demethoxygrandisin B	IC_50_ = 7.0 µM	IC_50_ = 26.04 µM	(CC_50_ = 26.04 µM) High selectivity, low toxicity	3.7	Mitochondrial damage, interaction with TryR
β-Sitosterol	IC_50_ = 17.7 ± 0.43 µg/mL	Induction of apoptosis	(>500 µg/mL) Low toxicity	>28.2	Increased ROS and mitochondrial depolarization
*Sterculia villosa* (methanolic extract)	IC_50_ = 17.5 µg/mL	DNA fragmentation	Low toxicity	ND	ROS overproduction and oxidative stress
Flavopereirine (*Geissospermum vellosii*)	IC_50_ = 0.23 µg/mL (24 h)	IC_50_ = 0.15 µg/mL (72 h)	(CC_50_ = 499.3 µg/mL) High selectivity	3328.7	Inhibition of oligopeptidase B
*Aspidosperma nitidum* (ethanol extract)	IC_50_ = 23.87 µg/mL	Reduction of parasite load in vivo	(CC_50_ = 500 µg/mL) in vitro No toxicity in vivo	21	Inhibition of trypanothione reductase, apoptosis
*Aspidosperma nitidum* (alkaloidal fraction)	IC_50_ = 18.5 µg/mL	Reduction of parasite load in vivo	(CC_50_ = 200 µg/mL) in vitro No toxicity in vivo	11	Inhibition of trypanothione reductase, apoptosis
Artemether (ART)	IC_50_ = 16.43 µg/mL	IC_50_ = 37.12 µg/mL	Reduced toxicity	ND	Interference in mitochondrial phosphorylation
Artemether (NLC-ART)	IC_50_ = 15.42 µg/mL	IC_50_ = 32.1 µg/mL	Reduced toxicity	ND	Interference in mitochondrial phosphorylation
Salidroside (*Rhodiola* spp.)	Reduction of promastigote growth	Reduction of parasite load	Low liver and kidney toxicity	ND	Modulation of the immune response, increased NO and ROS
Iridoid glycosides (*Nyctanthes arbortristis*)	Induction of apoptosis via oxidative stress	ROS-induced cell death	Low cytotoxicity in normal cells	ND	Mitochondrial oxidative damage, apoptosis
HO-3867 (Curcumin analogue)	Cell cycle arrest	Interruption of intracellular charge	Low cytotoxicity to macrophages	ND	Disruption of the STAT3 pathway and activation of apoptotic pathways

Legend: IC_50_: 50% inhibitory concentration; CC_50_: 50% cytotoxic concentration; ND: not determined; NO: nitric oxide; PARP1: Poly (ADP-ribose) Polymerase 1; ROS: reactive oxygen species; SI: selectivity index; STAT3: transcription factor involved in cell growth and survival regulation; TryR: trypanothione reductase.

## Data Availability

This review article did not use new data. The data related to this study are not available due to privacy restrictions or ethical considerations.

## Abbreviations

LC	Cutaneous leishmaniasis
LV	Visceral leishmaniasis
GLP	Glycosylphosphatidylinositol
CRP	C-reactive protein
CR4	Complement receptor 4
PKC	Protein kinase C
CL	Langerhans cells
LTA	American tegumentary leishmaniasis
NO	Nitric oxide
NOS2	Nitric oxide synthase type 2
ROS	Reactive oxygen species
O_2_^−^	Superoxide anions
H_2_O_2_	Hydrogen peroxide
NADPH oxidase	Nicotinamide Adenine Dinucleotide Phosphate Oxidase
O_2_	Molecular oxygen
RNS	Reactive nitrogen species
TXNPx	Thioredoxin peroxidase
FeSOD	Iron superoxide dismutase
MA	Meglumine antimoniate
ZnNPs	Zinc nanoparticles synthesized by green synthesis
CC_50_	50% cytotoxic concentration
HEVS	Hexane extract
SI	Selectivity index
IC_50_	50% inhibitory concentration
